# Development and validation of a multi-center nomogram for the presence of diabetic retinopathy in patients with type 2 diabetes: incorporating homocysteine, glycemic, lipid, and renal markers

**DOI:** 10.3389/fendo.2026.1822839

**Published:** 2026-04-22

**Authors:** Liming Wu, Risu Na, Ling Qiu

**Affiliations:** 1 Department of Endocrinology, Shanghai Fengxian District Central Hospital, Shanghai, China; 2 Department of Endocrinology, Shanghai Xuhui District Central Hospital, Shanghai, China

**Keywords:** diabetic retinopathy, homocysteine, nomogram, risk assessment tool, type 2 diabetes mellitus

## Abstract

**Objective:**

To develop and validate a nomogram that integrates plasma homocysteine with glycemic, lipid, and renal markers for estimating the probability of prevalent diabetic retinopathy (DR) in patients with type 2 diabetes mellitus (T2DM).

**Methods:**

This multi-center retrospective study included 930 patients. A development cohort (n=651; 145 DR events, 22.3% prevalence) was recruited from Shanghai Fengxian District Central Hospital, and an independent validation cohort (n=279; 71 DR events, 25.4% prevalence) was recruited from Shanghai Xuhui District Central Hospital. The primary outcome was the presence of DR based on fundus examination. Candidate clinical variables included demographic, clinical, and laboratory data. After pre-screening for multicollinearity, Least Absolute Shrinkage and Selection Operator (LASSO) regression was used to select informative indicators. A multivariable logistic regression model was built, and a nomogram was constructed. Model performance was evaluated by discrimination (area under the curve, AUC), calibration (Brier score, calibration-in-the-large [CITL]), and clinical utility (Decision Curve Analysis, DCA).

**Results:**

Eight variables were selected for the final model: Age, T2DM Duration, Systolic Blood Pressure, HbA1c, HDL-C, estimated glomerular filtration rate (eGFR), urinary albumin-to-creatinine ratio (UACR), and Homocysteine. The nomogram demonstrated favorable discrimination with an AUC of 0.865 in the development cohort and maintained a stable performance with an AUC of 0.842 in the validation cohort. Calibration was adequate in both sets (development Brier score 0.132, CITL 0.02; validation Brier score 0.148, CITL 0.15). DCA showed a positive net benefit for clinical decision-making across a wide range of threshold probabilities (10% to 75%). Subgroup analyses confirmed consistent performance across different patient demographics.

**Conclusion:**

A nomogram combining homocysteine with traditional clinical and renal markers shows promising capability in assessing the probability of prevalent DR in T2DM patients. While demonstrating potential applicability for screening prioritization, these retrospective findings require further prospective validation before the tool can be routinely implemented to guide clinical management.

## 1 Introduction

Diabetic retinopathy (DR), a microvascular complication of type 2 diabetes mellitus (T2DM), stands as a leading cause of preventable blindness among working-age adults globally (1). The prevalence of T2DM is rising at an alarming rate worldwide, and it is estimated that approximately one-third of individuals with diabetes have some form of DR, with a significant portion at risk of vision-threatening stages of the disease (2). This escalating public health crisis imposes a substantial burden on both individuals and healthcare systems. While regular ophthalmological screening is the cornerstone of DR management, its effectiveness is often hampered by low patient adherence, limited access, and high costs, highlighting the urgent need for more efficient risk stratification tools (3). Achieving this requires a deeper understanding of the complex pathogenesis of the disease.

The pathogenesis of DR is complex and multifactorial. Several established risk factors are known to contribute to its development and progression, including longer duration of diabetes, chronic hyperglycemia as measured by glycated hemoglobin (HbA1c), and elevated blood pressure (4). While these traditional factors are integral to risk assessment, they do not fully account for the variability in DR risk among patients, suggesting that other pathological pathways are involved. The systemic nature of diabetic microvascular complications supports the investigation of shared pathophysiological mechanisms, particularly between DR and diabetic nephropathy. This “common soil” hypothesis posits that markers of renal dysfunction, such as increased urinary albumin-to-creatinine ratio (UACR) and reduced estimated glomerular filtration rate (eGFR), may also serve as informative indicators of retinal microvascular damage (5, 6).

Recent evidence has also pointed towards the role of metabolic biomarkers that lie outside traditional glycemic and hypertensive pathways. One such promising marker is plasma homocysteine. Elevated levels of homocysteine, a sulfur-containing amino acid, have been linked to endothelial dysfunction, oxidative stress, and inflammation, all of which are critical mechanisms in the breakdown of the blood-retinal barrier and the development of retinal neovascularization characteristic of DR (7, 8). While traditional markers like HbA1c reflect chronic, long-term metabolic control, homocysteine serves as a direct indicator of active endothelial toxicity and microvascular inflammation (8). Therefore, integrating homocysteine is expected to add significant predictive value beyond established clinical parameters by capturing a distinct, concurrent pathophysiological axis of retinal damage. Despite growing evidence of its association with DR, homocysteine is not yet incorporated into routine clinical risk assessment, representing a potential gap in current risk assessment strategies.

Although several risk assessment models for DR exist, most rely predominantly on a narrow set of traditional glycemic and hypertensive parameters (9). They frequently fail to capture the synergistic risk contributed by renal microvascular dysfunction and novel metabolic indicators, leaving a critical knowledge gap in comprehensive, multidimensional risk profiling. Risk assessment tools, particularly visual tools like nomograms, offer a practical approach to bridge this translational gap. A nomogram is specifically the most appropriate presentation format for this purpose because it translates a complex multivariable mathematical algorithm into an intuitive, point-based graphical interface (10). This allows physicians to rapidly and accurately estimate individualized risk probabilities at the bedside without requiring specialized software, which is essential for fast-paced primary care and endocrinology settings. Therefore, this study aimed to develop and validate an integrated nomogram that combines these established risk factors with plasma homocysteine levels into a unified assessment framework. The resulting tool aims to offer a practical and consistent method for personalized risk stratification, potentially facilitating earlier and more targeted clinical interventions for patients with T2DM.

## 2 Methods

### 2.1 Study design

This multi-center, retrospective observational study was designed to develop and externally validate a clinical nomogram for assessing the presence of DR in patients with T2DM. We retrospectively collected data from the electronic medical records (EMR) of T2DM patients who were evaluated in both the specialized endocrinology outpatient clinics and inpatient wards at Shanghai Fengxian District Central Hospital (Center A) and Shanghai Xuhui District Central Hospital (Center B) between January 1, 2022, and January 1, 2025. Both institutions are comprehensive tertiary care centers that strictly adhere to the standardized clinical pathways and management guidelines mandated by the Chinese Diabetes Society, ensuring highly comparable routine laboratory testing, including standardized homocysteine assays, and identical comprehensive fundus screening practices across both settings. The study protocol was comprehensively reviewed and received full approval from the Institutional Review Boards (IRB) of both Shanghai Fengxian District Central Hospital (Approval No. 2026-012) and Shanghai Xuhui District Central Hospital. Given the strictly retrospective observational nature of the study and the exclusive use of fully de-identified electronic medical records, the respective IRBs formally granted a waiver for individual patient informed consent. All procedures were conducted in strict adherence to the ethical principles outlined in the 1964 Declaration of Helsinki and its subsequent amendments. Furthermore, to ensure transparent and complete reporting of our prediction model development and validation process, this manuscript strictly adheres to the Transparent Reporting of a multivariable prediction model for Individual Prognosis Or Diagnosis (TRIPOD) statement.

### 2.2 Study population

The study cohort was retrospectively identified through a systematic query of the institutional EMR systems at both participating centers. The query targeted patients with an International Classification of Diseases, Tenth Revision (ICD-10) diagnosis code for Type 2 Diabetes Mellitus (E11) who had at least one visit. This initial automated screening was followed by a detailed manual review of each patient’s full medical record, conducted by two independent, trained clinical researchers, to confirm eligibility. Any discrepancies between the reviewers were resolved by a senior endocrinologist to ensure diagnostic accuracy and adherence to the study protocol. For model development and validation, the total cohort was divided by institutional origin. Patients from Shanghai Fengxian District Central Hospital (n=651) were assigned to the development cohort to construct the predictive nomogram. Patients from Shanghai Xuhui District Central Hospital (n=279) served as an independent external validation cohort to rigorously evaluate the model’s geographic and institutional generalizability. The inclusion and exclusion criteria were applied consistently across both centers.

Inclusion criteria were as follows: (1) adult patients (aged ≥ 18 years); (2) a confirmed diagnosis of T2DM according to the 2024 American Diabetes Association (ADA) “Standards of Care in Diabetes” criteria (11); (3) availability of a definitive fundus examination result, explicitly defined as high-quality, bilateral, 45-degree color fundus photographs centered on the macula and optic disc that were fully gradable by two independent specialists for the clear diagnosis and classification of DR; and (4) complete baseline data for all core laboratory variables, including homocysteine and HbA1c, obtained within a predefined window of 30 days prior to or following the definitive fundus examination.

Exclusion criteria were any of the following: (1) a diagnosis of type 1 diabetes, gestational diabetes, or other specific secondary types of diabetes; (2) presence of co-existing ocular diseases that could confound the diagnosis of DR, such as retinal vein occlusion, age-related macular degeneration, high-myopic retinopathy, or glaucoma, as detailed in the American Academy of Ophthalmology’s “Diabetic Retinopathy Preferred Practice Pattern^®^” (12); (3) severe hepatic dysfunction, defined as alanine or aspartate aminotransferase levels greater than three times the upper limit of normal, or the presence of non-diabetic chronic kidney disease (explicitly defined as biopsy-proven primary glomerulopathies, polycystic kidney disease, or obstructive nephropathy); and (4) a history of malignant tumors or other severe life-limiting conditions (such as end-stage heart failure [NYHA class IV] or severe chronic obstructive pulmonary disease) with a documented expected survival of less than one year.

The required sample size was estimated based on the “events per variable” (EPV) rule, a widely accepted standard for prediction model development. To ensure the stability and reliability of the final model, a minimum of 10 events (patients with DR) per potential candidate variable is recommended. Considering that LASSO regression might identify up to 15 significant predictors for the final model, a minimum of 150 DR events was deemed necessary (10 EPV × 15 variables). Based on a global DR prevalence of 22.27% reported in a large-scale, recent meta-analysis published in Ophthalmology (1), the minimum total sample size required to achieve this was calculated to be approximately 674 patients (150/0.2227). Therefore, our available cohort of approximately 900 patients is adequately powered for this study.

### 2.3 Data collection and variable definitions

All patient data were retrospectively collected from the institutional EMR system, which integrates the hospital information system (HIS), picture archiving and communication system (PACS), and laboratory information system (LIS). To ensure data accuracy and consistency, two trained researchers independently extracted information using a standardized data collection form. Any discrepancies were resolved by a third senior investigator to maintain data integrity. All clinical variables were based on the patient’s baseline status, defined as the data recorded during their initial hospital admission or first outpatient visit within the study period.

The primary outcome was the presence of DR, treated as a binary variable (DR vs. non-DR). The diagnosis was established based on a review of fundus photography (captured using a Topcon TRC-NW8 non-mydriatic retinal camera; Topcon, Tokyo, Japan) or fluorescein angiography (FFA) images by two senior ophthalmologists who were blinded to the patients’ clinical and laboratory data. Diagnoses were graded according to the International Clinical Diabetic Retinopathy Disease Severity Scale (13). Patients with any stage of DR (mild, moderate, or severe non-proliferative DR, or proliferative DR) were categorized into the DR group, while those without any detectable abnormalities were assigned to the non-DR group. Cases with diagnostic disagreement were adjudicated by a third, more senior retinal specialist.

Predictor variables were comprehensively documented based on the patient’s baseline status, encompassing demographic, anthropometric, laboratory, and historical data. Demographic and baseline characteristics included age, sex, duration of T2DM, smoking history (current/former/never), and alcohol consumption (yes/no). Anthropometric and vital sign measurements consisted of body mass index (BMI), calculated as weight in kilograms divided by the square of height in meters (kg/m²), systolic blood pressure (SBP), and diastolic blood pressure (DBP). A full panel of laboratory parameters was systematically collected from fasting venous blood and morning urine samples. To ensure measurement comparability across both centers, all biochemical analyses were performed using standardized automated clinical chemistry analyzers (Cobas 8000; Roche Diagnostics, Mannheim, Germany) subject to rigorous external quality assessment. Fasting plasma glucose (FPG) and a complete lipid profile (total cholesterol [TC], triglycerides [TG], low-density lipoprotein cholesterol [LDL-C], and high-density lipoprotein cholesterol [HDL-C]) were measured via enzymatic colorimetric methods utilizing the standardized automated clinical chemistry analyzers (Cobas 8000; Roche Diagnostics, Mannheim, Germany). Glycated hemoglobin (HbA1c) was strictly determined using high-performance liquid chromatography (HPLC) on a dedicated analyzer (D-100 System; Bio-Rad, Hercules, CA, USA). Specifically, serum creatinine [Scr] was determined via an isotope dilution mass spectrometry (IDMS)-traceable enzymatic method to ensure precise calculation of the estimated glomerular filtration rate [eGFR] (calculated using the 2021 Chronic Kidney Disease Epidemiology Collaboration (CKD-EPI) equation without the race variable (14)), and the urinary albumin-to-creatinine ratio [UACR] was quantified using immunoturbidimetry. The core study marker, plasma homocysteine (Hcy), was measured using a highly sensitive enzymatic cycling assay with commercially available diagnostic kits (Maccura Biotechnology Co., Ltd., Chengdu, China) on the Cobas 8000 platform. Crucially, all laboratory tests were obtained within a predefined window of 30 days prior to or following the definitive fundus examination to guarantee an adequate temporal correlation between systemic biomarkers and retinal status while accounting for retrospective clinical workflows. Furthermore, comorbidities and baseline medication use were recorded as binary variables based on documented diagnoses, ICD-10 codes, or medication records in the EMR.

### 2.4 Data quality control

To ensure the reliability of the primary outcome assessment and minimize potential diagnostic bias, a rigorous data quality control protocol was implemented. The diagnosis of DR, our primary outcome, was determined through an independent and blinded review process. Two senior ophthalmologists, each with over 10 years of experience in retinal diseases and blinded to all patient clinical and laboratory data, independently evaluated all fundus photographs and FFA images. To formally quantify the level of agreement between the two specialists, inter-observer reliability was assessed in a randomly selected subsample of 100 patients. The analysis yielded a Cohen’s Kappa coefficient of 0.91 (95% CI: 0.85-0.97), indicating almost perfect agreement in the diagnosis of DR. Any diagnostic discrepancies between the two primary reviewers were resolved by a third, more senior retinal specialist, whose decision was final. Furthermore, to ensure the accuracy of the predictor variable data, a random 10% sample of the electronic medical records was independently re-extracted and cross-verified by a different researcher, with a discrepancy rate of less than 1% found.

### 2.5 Statistical analysis

Statistical methodologies and data analyses were conducted under the formal consultation and rigorous review of an independent biostatistician from our institutional clinical research center. All statistical analyses were performed using R software (version 4.2.2; R Foundation for Statistical Computing, Vienna, Austria), and a two-sided P-value < 0.05 was considered statistically significant. Continuous variables were presented as mean ± standard deviation (SD) for normally distributed data or median with interquartile range (IQR) for skewed data, while categorical variables were summarized as frequency counts and percentages (n, %). As per our inclusion criteria, complete datasets were available for core variables, including age, sex, HbA1c, and homocysteine (0% missing). Missing data were only present in a few non-core variables and were minimal (<5%): body mass index (2.1%), T2DM duration (1.5%), HDL-C (1.8%), and LDL-C (2.5%). Assuming a missing at random (MAR) mechanism, these specific missing values were handled using multiple imputation by chained equations (MICE). To rigorously prevent data leakage, the imputation procedure was performed strictly separately within the development and external validation datasets. For each dataset, we generated five imputed datasets. The imputation models incorporated all candidate predictor variables alongside the primary outcome variable (diabetic retinopathy) to ensure robust and unbiased estimates. In the training set, baseline characteristics between patients with and without DR were compared using the Student’s t-test or Mann-Whitney U test for continuous variables, and the Chi-square test or Fisher’s exact test for categorical variables. Prior to model building, we conducted a formal statistical assessment to detect multicollinearity among all candidate variables by calculating the variance inflation factor (VIF). Variables demonstrating a VIF > 5 were considered to have significant collinearity. This formal mathematical assessment perfectly aligned with our clinical rationale: serum creatinine (highly collinear with eGFR, VIF > 5), history of diabetic nephropathy (collinear with UACR and eGFR, VIF > 5), and history of hypertension (collinear with systolic blood pressure, VIF > 5) exhibited problematic collinearity. Consequently, to ensure model stability and avoid redundancy, these specific variables were pre-excluded. The remaining 22 candidate predictor variables were then subjected to a Least Absolute Shrinkage and Selection Operator (LASSO) logistic regression model in the training set to identify the most informative associated indicators. The optimal penalty parameter (lambda, λ) was determined via 10-fold cross-validation based on binomial deviance, which is appropriate for a binary outcome. To construct a more parsimonious model and rigorously prevent overfitting, we utilized the one-standard-error (1-SE) rule rather than the absolute minimum deviance. Variables with non-zero coefficients at this 1-SE optimal lambda were selected for the multivariable logistic regression model. Prior to fitting the final model, we utilized restricted cubic splines (RCS) with three knots to formally evaluate potential non-linear relationships between the continuous predictors and the log-odds of diabetic retinopathy. To determine the optimal functional form, models with linear terms were compared to models incorporating RCS using likelihood ratio tests and Akaike Information Criterion (AIC). To evaluate internal validity and quantify potential model overfitting within the development phase, we performed internal validation using 1000 bootstrap resamples in the training cohort to calculate the optimism-corrected C-index. Subsequently, the performance and generalizability of the nomogram were rigorously evaluated in the independent external validation set. Its discrimination was assessed by calculating the area under the receiver operating characteristic curve (AUC), also known as the C-index. Calibration was comprehensively evaluated not only using visual calibration plots and the Hosmer-Lemeshow test but also through modern quantitative metrics recommended by the TRIPOD guidelines: the Brier score to assess overall predictive accuracy, calibration-in-the-large (CITL) to detect systematic over- or under-estimation, and the calibration slope to evaluate the spread of predicted risks. For the training cohort, these calibration metrics were optimism-corrected alongside the C-index. To determine the clinical utility of the nomogram, Decision Curve Analysis (DCA) was conducted. Furthermore, to rigorously quantify the incremental predictive value of homocysteine, we compared the full model against a base clinical model (excluding homocysteine). This was evaluated by calculating the change in the C-index (ΔAUC) using DeLong’s test, the continuous Net Reclassification Improvement (NRI), and the Integrated Discrimination Improvement (IDI). Finally, to assess the model’s stability across diverse patient profiles, subgroup analyses were performed. To ensure adequate statistical power for detecting potential heterogeneities, these analyses were conducted utilizing the pooled sample (N = 930). Interaction testing was formally evaluated by introducing an interaction term between the model’s calculated linear predictor and each respective stratifying variable within the logistic regression framework. To accurately reflect the original model’s true unadjusted transportability, the discriminative performance (C-index) within each subgroup was calculated without any recalibration, and the results were visualized using forest plots.

## 3 Results

### 3.1 Patient selection and baseline characteristics

**Figure 1:**
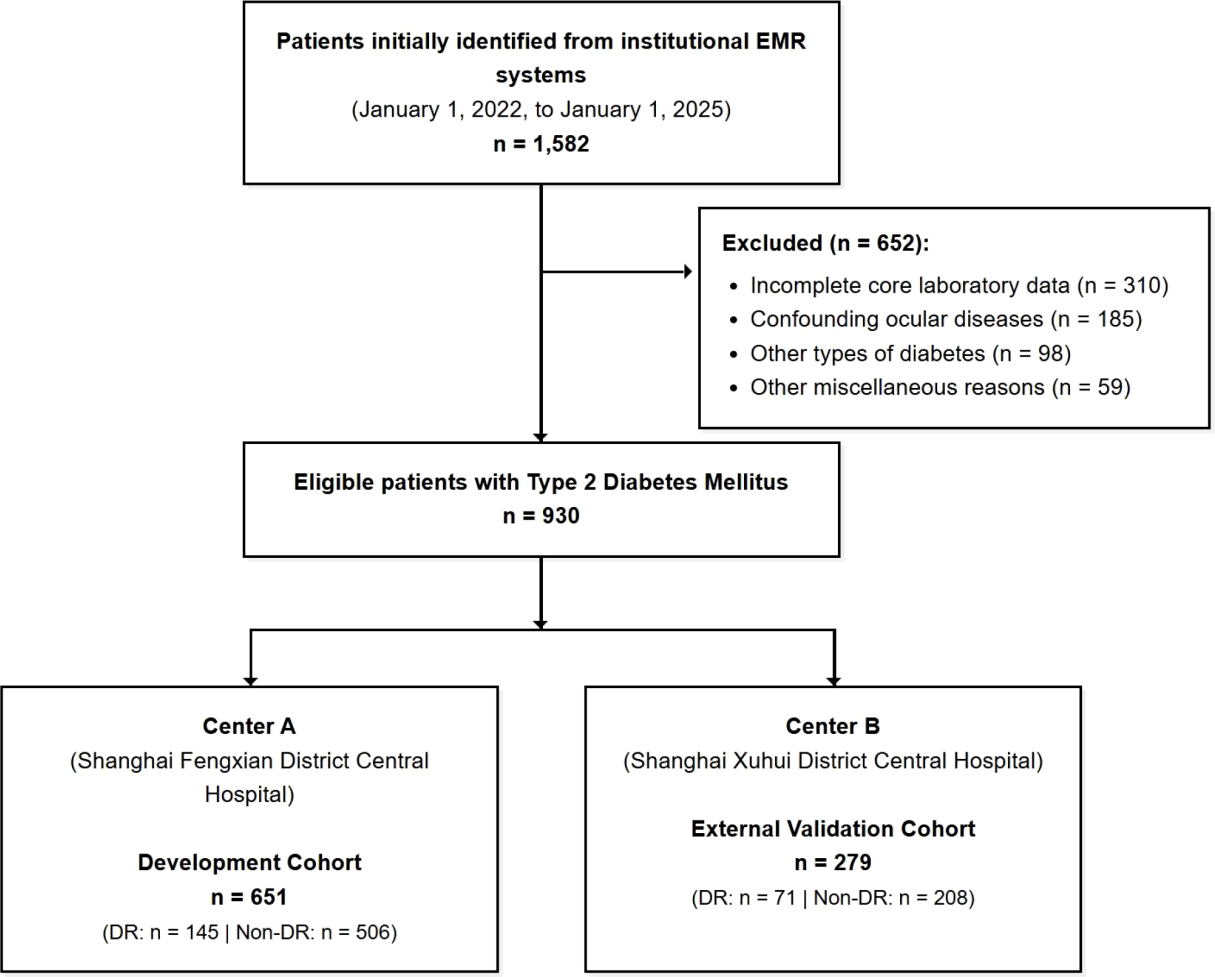
Participant flow diagram for the study cohort.

A total of 1,582 patients with a T2DM diagnosis were initially identified from the electronic medical record system. After applying the predefined inclusion and exclusion criteria, 652 patients were excluded for reasons including the presence of confounding ocular diseases (n=185), incomplete core laboratory data (n=310), a diagnosis of other types of diabetes (n=98), and other miscellaneous reasons (n=59). To address potential selection bias, we formally compared the baseline characteristics of the 930 included patients with the 310 patients excluded specifically due to missing core laboratory data (such as homocysteine). As detailed in **Supplementary Table S1**, there were no clinically or statistically significant differences in major demographic or metabolic parameters between the two groups, suggesting that the exclusion process did not introduce substantial systematic bias into our final cohort. Ultimately, a cohort of 930 eligible patients was included in the final analysis. Based on their institutional origin, these patients were assigned to a training set (n=651, from Center A) for model development and an independent external validation set (n=279, from Center B) to evaluate the model’s generalizability (**Figure 1**). The baseline demographic, clinical, and laboratory characteristics of the training and validation sets are summarized in **Table 1**. No statistically significant differences were observed between the two sets for any of the variables (all P > 0.05), indicating highly comparable patient populations across the two independent clinical centers. Within the training cohort, 145 of the 651 patients (22.3%) were diagnosed with DR. In the independent external validation cohort, 71 of the 279 patients (25.4%) experienced DR events, reflecting a realistic natural variation between the two clinical centers. This yielded a total of 216 DR events across the entire study population (N = 930, overall prevalence 23.2%). The detailed comparison of baseline characteristics between patients with and without DR in the training set is presented in **Table 2**. Patients in the DR group had a significantly longer duration of T2DM, higher levels of SBP, HbA1c, FPG, TG, UACR, and Hcy, and a higher prevalence of hypertension, diabetic nephropathy, diabetic peripheral neuropathy, and antidiabetic drug use. Conversely, the DR group exhibited significantly lower levels of HDL-C and eGFR compared to the non-DR group (all P < 0.05).

**Table 1:** Baseline characteristics of patients in the training and validation cohorts.

Characteristic	Training set (n=651)	Validation set (n=279)	Statistic	P-value
Demographics				
Age, years	59.8 ± 10.1	60.1 ± 9.9	t=-0.41	0.681
Sex, Male, n (%)	366 (56.2)	159 (57.0)	χ²=0.05	0.829
T2DM Duration, years	8.0 (5.0-12.0)	7.0 (5.0-13.0)	U=90214	0.855
Smoking history, n (%)			χ²=0.25	0.881
Never	401 (61.6)	174 (62.4)		
Former/Current	250 (38.4)	105 (37.6)		
Alcohol consumption, Yes, n (%)	205 (31.5)	85 (30.5)	χ²=0.11	0.738
Anthropometric & Vitals				
Body mass index, kg/m²	26.1 ± 3.8	26.3 ± 4.0	t=-0.73	0.467
Systolic blood pressure, mmHg	135.4 ± 18.2	134.9 ± 17.9	t=0.38	0.701
Diastolic blood pressure, mmHg	81.1 ± 10.5	80.8 ± 10.2	t=0.39	0.694
Laboratory Parameters				
HbA1c, %	7.5 ± 1.4	7.6 ± 1.5	t=-0.91	0.363
Fasting plasma glucose, mmol/L	8.1 ± 2.4	8.2 ± 2.6	t=-0.58	0.562
Total cholesterol, mmol/L	4.6 ± 1.1	4.7 ± 1.0	t=-1.25	0.213
Triglycerides, mmol/L	1.8 (1.2-2.6)	1.7 (1.3-2.5)	U=91055	0.988
LDL-C, mmol/L	2.8 ± 0.9	2.7 ± 0.8	t=1.51	0.131
HDL-C, mmol/L	1.1 ± 0.3	1.1 ± 0.3	t=-0.37	0.710
Serum creatinine, μmol/L	75.0 (65.0-88.0)	74.0 (66.0-87.0)	U=89987	0.781
eGFR, ml/min/1.73m²	92.5 ± 22.8	93.1 ± 23.5	t=-0.38	0.704
UACR, mg/g	25.0 (14.0-58.0)	26.0 (15.0-55.0)	U=90543	0.901
Homocysteine, μmol/L	12.3 ± 4.4	12.1 ± 4.1	t=0.66	0.510
Comorbidities & Medication History				
Hypertension, n (%)	352 (54.1)	148 (53.0)	χ²=0.08	0.772
Dyslipidemia, n (%)	301 (46.2)	125 (44.8)	χ²=0.15	0.699
Cardiovascular disease, n (%)	96 (14.7)	43 (15.4)	χ²=0.07	0.796
Diabetic Nephropathy, n (%)	182 (28.0)	78 (28.0)	χ²=0.00	0.998
Diabetic Peripheral Neuropathy, n (%)	209 (32.1)	89 (31.9)	χ²=0.00	0.957
Use of antidiabetic drugs, n (%)	529 (81.3)	225 (80.6)	χ²=0.05	0.821
Use of antihypertensive drugs, n (%)	340 (52.2)	141 (50.5)	χ²=0.22	0.641
Use of lipid-lowering drugs, n (%)	289 (44.4)	128 (45.9)	χ²=0.18	0.668

Data are presented as mean ± standard deviation, median (interquartile range), or n (%). P-values were calculated using Student’s t-test for normally distributed continuous variables, Mann-Whitney U test for non-normally distributed continuous variables, and the Chi-square test for categorical variables. T2DM, type 2 diabetes mellitus; HbA1c, glycated hemoglobin; LDL-C, low-density lipoprotein cholesterol; HDL-C, high-density lipoprotein cholesterol; eGFR, estimated glomerular filtration rate; UACR, urinary albumin-to-creatinine ratio.

**Table 2:** Comparison of baseline characteristics between patients with and without diabetic retinopathy in the training cohort (n=651).

Characteristic	Non-DR group (n=506)	DR group (n=145)	Statistic	P-value
Demographics				
Age, years	59.2 ± 10.3	61.8 ± 9.1	t=-2.72	0.007
Sex, Male, n (%)	281 (55.5)	85 (58.6)	χ²=0.44	0.506
T2DM Duration, years	6.0 (4.0-10.0)	13.0 (9.0-18.0)	U=15321	<0.001
Smoking history, n (%)			χ²=1.98	0.159
Never	318 (62.8)	83 (57.2)		
Former/Current	188 (37.2)	62 (42.8)		
Alcohol consumption, Yes, n (%)	155 (30.6)	50 (34.5)	χ²=0.84	0.359
Anthropometric & Vitals				
Body mass index, kg/m²	26.2 ± 3.9	25.8 ± 3.4	t=1.15	0.251
Systolic blood pressure, mmHg	132.8 ± 16.9	144.5 ± 19.8	t=-6.78	<0.001
Diastolic blood pressure, mmHg	80.5 ± 10.1	83.3 ± 11.2	t=-2.79	0.005
Laboratory Parameters				
HbA1c, %	7.2 ± 1.1	8.5 ± 1.8	t=-9.11	<0.001
Fasting plasma glucose, mmol/L	7.7 ± 2.1	9.5 ± 2.9	t=-7.35	<0.001
Total cholesterol, mmol/L	4.6 ± 1.0	4.7 ± 1.2	t=-0.98	0.327
Triglycerides, mmol/L	1.7 (1.2-2.4)	2.1 (1.5-3.1)	U=26815	<0.001
LDL-C, mmol/L	2.8 ± 0.9	2.8 ± 1.0	t=-0.21	0.837
HDL-C, mmol/L	1.2 ± 0.3	1.0 ± 0.2	t=8.15	<0.001
Serum creatinine, μmol/L	72.0 (64.0-84.0)	85.0 (72.0-101.0)	U=23955	<0.001
eGFR, ml/min/1.73m²	95.8 ± 21.1	81.3 ± 24.5	t=6.82	<0.001
UACR, mg/g	21.0 (12.0-35.0)	65.0 (40.0-155.0)	U=12980	<0.001
Homocysteine, μmol/L	11.2 ± 3.5	16.1 ± 4.9	t=-12.11	<0.001
Comorbidities & Medication History				
Hypertension, n (%)	249 (49.2)	103 (71.0)	χ²=23.15	<0.001
Dyslipidemia, n (%)	230 (45.5)	71 (49.0)	χ²=0.61	0.435
Cardiovascular disease, n (%)	65 (12.8)	31 (21.4)	χ²=6.93	0.008
Diabetic Nephropathy, n (%)	95 (18.8)	87 (60.0)	χ²=116.35	<0.001
Diabetic Peripheral Neuropathy, n (%)	115 (22.7)	94 (64.8)	χ²=122.01	<0.001
Use of antidiabetic drugs, n (%)	401 (79.2)	128 (88.3)	χ²=7.51	0.006
Use of antihypertensive drugs, n (%)	241 (47.6)	99 (68.3)	χ²=19.97	<0.001
Use of lipid-lowering drugs, n (%)	215 (42.5)	74 (51.0)	χ²=3.19	0.074

Data are presented as mean ± standard deviation, median (interquartile range), or n (%). P-values were calculated using Student’s t-test for normally distributed continuous variables, Mann-Whitney U test for non-normally distributed continuous variables, and the Chi-square test for categorical variables. DR, diabetic retinopathy; T2DM, type 2 diabetes mellitus; HbA1c, glycated hemoglobin; LDL-C, low-density lipoprotein cholesterol; HDL-C, high-density lipoprotein cholesterol; eGFR, estimated glomerular filtration rate; UACR, urinary albumin-to-creatinine ratio.

### 3.2 Variable selection by LASSO regression

**Figure 2:**
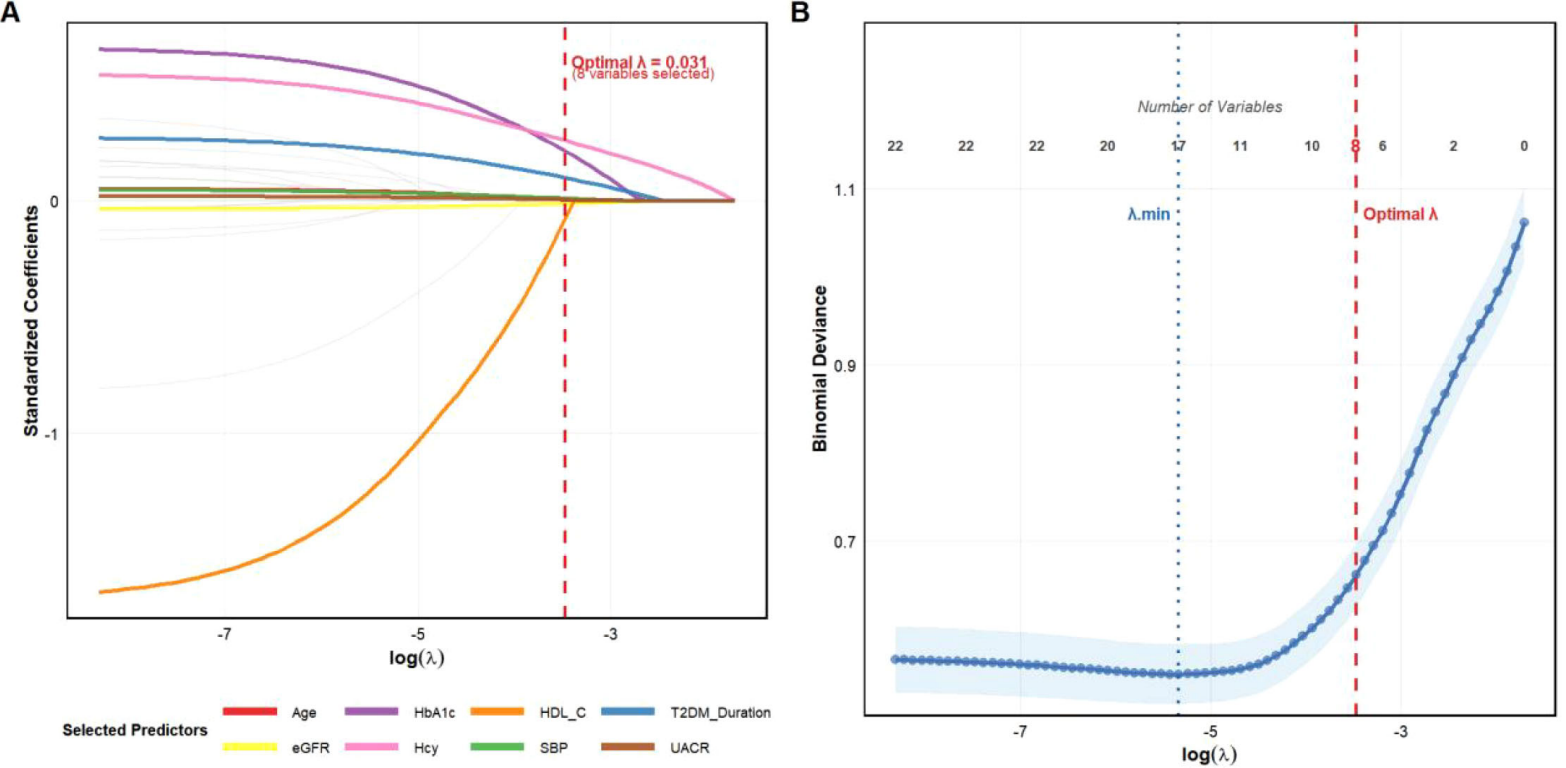
LASSO regression analysis for variable selection in diabetic retinopathy prediction model. **(A)** LASSO coefficient profiles of the 22 candidate predictor variables plotted against the log(λ) sequence. Each colored line represents the trajectory of a standardized coefficient for one variable as the penalty parameter (λ) increases from left to right. The vertical red dashed line indicates the optimal λ value (λ = 0.031, log(λ) = -3.47) selected for the final model, at which point 8 variables retained non-zero coefficients. Variables compressed to zero (gray lines) were excluded from the model. The eight selected predictors are highlighted in distinct colors: Age (red), HbA1c (purple), HDL-C (orange), T2DM Duration (blue), eGFR (yellow), Homocysteine (pink), Systolic Blood Pressure (green), and UACR (brown). **(B)** Ten-fold cross-validation curve for tuning parameter selection in the LASSO model. The blue curve represents the binomial deviance (y-axis) plotted against log(λ) values (x-axis), with error bars indicating ± 1 standard error (shaded region). The blue dotted vertical line marks λ.min (the λ value that gives minimum mean cross-validated error), while the red dashed vertical line indicates the optimal λ = 0.031 selected for this study. Numbers along the top axis denote the number of variables with non-zero coefficients at each corresponding λ value. At the optimal λ, 8 variables were retained in the model, balancing model complexity and predictive performance. LASSO, Least Absolute Shrinkage and Selection Operator; λ, penalty parameter; HbA1c, glycated hemoglobin; HDL-C, high-density lipoprotein cholesterol; T2DM, type 2 diabetes mellitus; eGFR, estimated glomerular filtration rate; UACR, urinary albumin-to-creatinine ratio; SBP, systolic blood pressure.

**Figure 3:**
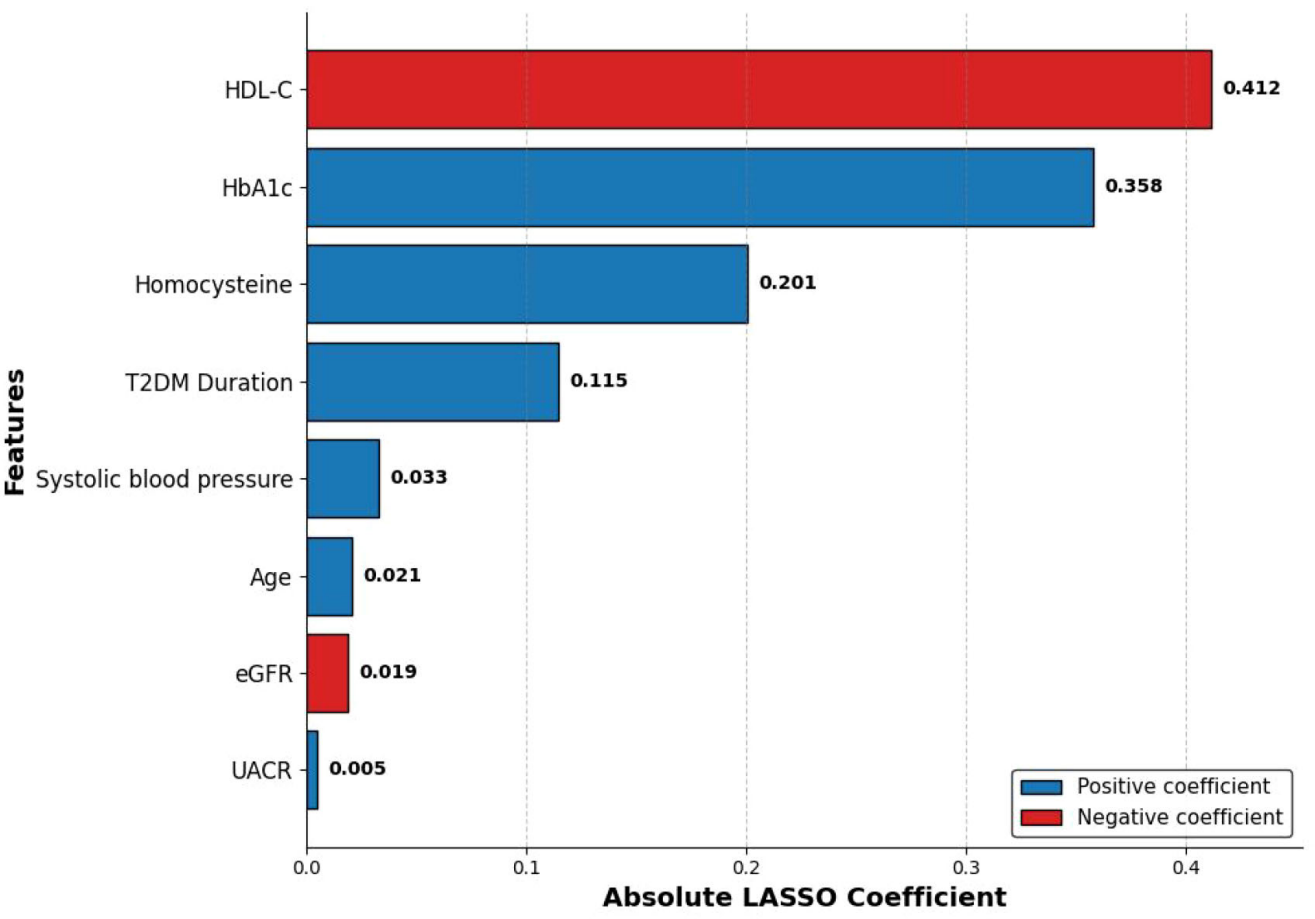
Relative importance ranking of the eight predictors selected by the LASSO model. This bar plot visually ranks the eight variables from most to least important based on the absolute value of their LASSO coefficients, clearly highlighting HDL-C and HbA1c as the most significant predictors.

Following the pre-screening step to reduce multicollinearity, a total of 22 candidate predictor variables were entered into the LASSO regression model for further selection. Through the process of 10-fold cross-validation in the training cohort, the tuning parameter was evaluated using binomial deviance. By applying the 1-SE rule to prioritize model parsimony over the absolute minimum deviance (λ.min, which would have retained 17 variables), the optimal penalty parameter was determined to be λ = 0.031 (**Figure 1B**). At this optimal λ value, the coefficients of 14 variables were compressed to exactly zero, leaving a concise panel of eight variables with non-zero coefficients as the most informative indicators of prevalent DR (**Figures 2A, B**). These selected predictors included Age, T2DM Duration, Systolic blood pressure, HbA1c, HDL-C, eGFR, UACR, and Homocysteine. To better visualize the relative contribution of each selected predictor within the LASSO model, their importance was ranked based on the absolute value of their regression coefficients. This analysis revealed that HDL-C, HbA1c, and Homocysteine were the top three most influential predictors identified at this stage (**Figure 3**).

### 3.3 Development of the risk assessment model for DR

Prior to constructing the final multivariable model, the linearity assumption for all selected continuous predictors was formally assessed using restricted cubic splines (**Supplementary Figure S1**). The formal non-linearity tests indicated that Age, T2DM Duration, HDL-C, eGFR, UACR, and Homocysteine exhibited strictly linear relationships with the log-odds of DR (all P for non-linearity > 0.05). While mild non-linear deviations were observed at the extreme data tails for HbA1c and Systolic Blood Pressure (P for non-linearity < 0.05), visual inspection confirmed that their risk trajectories remained predominantly monotonic across the clinically relevant interquartile ranges. Furthermore, likelihood ratio tests comparing the simple linear models with the complex RCS-transformed models revealed no significant improvement in overall predictive performance (P > 0.05 for LRT, with negligible changes in AIC and C-index). Therefore, to prioritize clinical interpretability, model parsimony, and the practical utility of the visual nomogram, all continuous variables were pragmatically retained as simple linear terms in the final multivariable logistic regression model. The results, including the regression coefficients (β), odds ratios (ORs), 95% confidence intervals (CIs), and corresponding P-values for each predictor, are detailed in **Table 3**. All eight variables remained statistically significant predictors of DR in the multivariable analysis (all P < 0.05), confirming their roles as independent risk or protective factors after adjusting for confounders.

**Table 3:** Multivariable logistic regression analysis of predictors for diabetic retinopathy in the training cohort.

Predictor	β	SE	Wald	OR (95% CI)	P-value
Age (per year)	0.045	0.021	4.58	1.05 (1.01-1.09)	0.032
T2DM Duration (per year)	0.174	0.035	24.60	1.19 (1.11-1.28)	<0.001
Systolic blood pressure (per mmHg)	0.031	0.009	11.91	1.03 (1.01-1.05)	<0.001
HbA1c (per 1%)	0.445	0.088	25.54	1.56 (1.31-1.86)	<0.001
HDL-C (per mmol/L)	-0.635	0.281	5.09	0.53 (0.31-0.92)	0.024
eGFR (per ml/min/1.73m²)	-0.022	0.006	13.62	0.98 (0.97-0.99)	<0.001
UACR (per 10 mg/g)	0.041	0.012	11.76	1.04 (1.02-1.07)	<0.001
Homocysteine (per μmol/L)	0.183	0.033	30.58	1.20 (1.13-1.28)	<0.001

The multivariable logistic regression model was built using the training cohort data (n=651). β, regression coefficient; SE, standard error; OR, odds ratio; CI, confidence interval; T2DM, type 2 diabetes mellitus; HbA1c, glycated hemoglobin; HDL-C, high-density lipoprotein cholesterol; eGFR, estimated glomerular filtration rate; UACR, urinary albumin-to-creatinine ratio.

**Figure 4:**
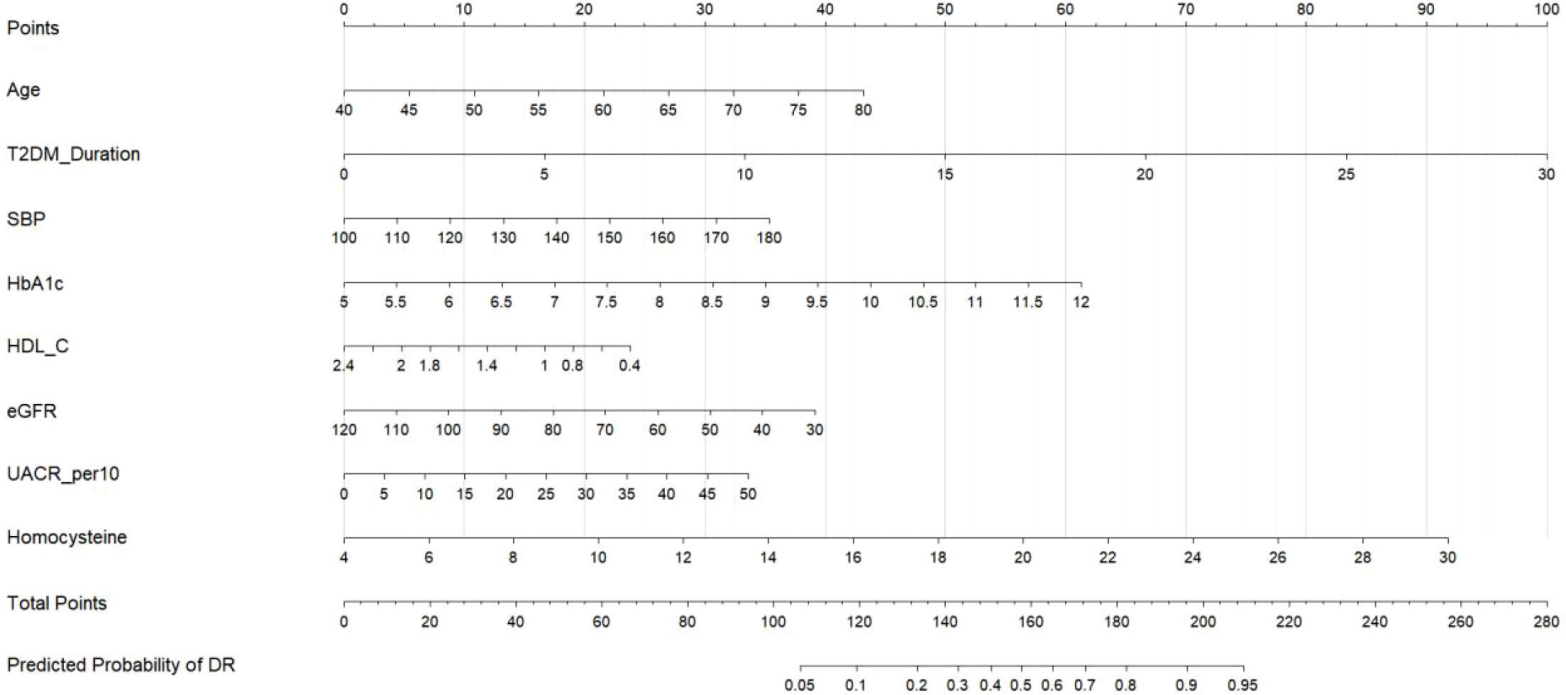
Nomogram for estimating the probability of prevalent diabetic retinopathy. This nomogram integrates eight clinical predictors for individualized risk assessment of diabetic retinopathy in patients with type 2 diabetes mellitus. Each predictor variable is displayed on a separate horizontal axis with its measurement scale. To calculate an individual patient’s risk, locate the patient’s value on each predictor axis, draw a vertical line upward to the “Points” axis to determine the corresponding points, sum all points to obtain a “Total Points” score, and project this total score downward to the “Predicted Probability of DR” axis to read the estimated risk probability. The eight predictors included are: Age (years), T2DM Duration (years), Systolic Blood Pressure (mmHg), HbA1c (%), HDL-C (mmol/L), eGFR (ml/min/1.73m²), UACR (per 10 mg/g), and Homocysteine (μmol/L). T2DM, type 2 diabetes mellitus; HbA1c, glycated hemoglobin; HDL-C, high-density lipoprotein cholesterol; eGFR, estimated glomerular filtration rate; UACR, urinary albumin-to-creatinine ratio; DR, diabetic retinopathy.

To facilitate clinical application and individualized risk assessment, a nomogram was developed based on this final multivariable model (**Figure 4**). This graphical tool allows clinicians to easily determine the risk of DR for an individual patient. By locating a patient’s value for each of the eight predictors on its corresponding axis, assigning the indicated points, and summing these to a “Total Points” score, one can directly read the corresponding estimated probability of prevalent diabetic retinopathy on the final risk scale.

To illustrate the clinical application of the nomogram, consider a hypothetical 68-year-old male patient (≈ 25 points) with a 15-year history of T2DM (≈ 50 points). His recent clinical measurements show a systolic blood pressure of 145 mmHg (≈ 20 points) and an HbA1c of 9.2% (≈ 60 points). His lipid profile reveals a low HDL-C of 0.9 mmol/L (≈ 45 points), and renal function tests indicate an eGFR of 75 ml/min/1.73m² (≈ 28 points) and a UACR of 35 mg/g (≈ 15 points). His serum homocysteine level is measured at 17 µmol/L (≈ 55 points). Summing these values yields a total of 298 points. By locating this total score on the nomogram, the patient’s predicted risk of having diabetic retinopathy is approximately 65%. In clinical practice, this individual probability score allows clinicians to assess the patient’s risk on a continuous scale, facilitating personalized screening decisions. This quantitative risk assessment would prompt the clinical team to strongly recommend an immediate referral for a comprehensive ophthalmological examination, intensify management of his glycemic control and blood pressure, and potentially consider lifestyle and therapeutic interventions to address his elevated homocysteine and low HDL-C levels.

Furthermore, to facilitate independent validation and exact calculation of the predicted risk without solely relying on the visual nomogram, the full mathematical equation of the model is explicitly provided. The predicted probability (P) of having diabetic retinopathy can be calculated using the logistic transformation formula: P = 1/(1 + exp(-Z)), where the linear predictor Z is calculated as follows: Z = -12.450 + 0.045 * Age (years) + 0.174 * T2DM Duration (years) + 0.031 * Systolic blood pressure (mmHg) + 0.445 * HbA1c (%) - 0.635 * HDL-C (mmol/L) - 0.022 * eGFR (ml/min/1.73m2) + 0.041 * UACR (per 10 mg/g) + 0.183 * Homocysteine (μmol/L).

### 3.4 Model performance, validation, and clinical utility

**Figure 5:**
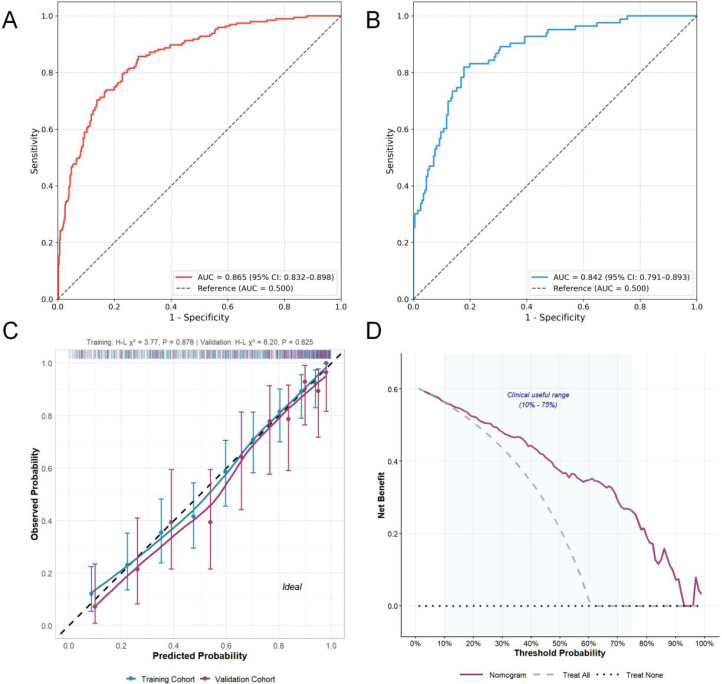
Nomogram performance evaluation. Performance metrics of the diabetic retinopathy prediction nomogram in training and validation cohorts. **(A, B)** Receiver operating characteristic (ROC) curves demonstrating excellent discrimination in both training (AUC = 0.865, 95% CI: 0.832–0.898) and validation (AUC = 0.842, 95% CI: 0.791–0.893) cohorts. The dashed diagonal line represents random chance (AUC = 0.500). **(C)** Calibration curves showing strong agreement between predicted probabilities and observed outcomes in both cohorts. Points represent deciles of predicted risk with 95% confidence intervals. The dashed diagonal line indicates perfect calibration. Rug plots show predicted probability distributions. Hosmer-Lemeshow tests confirmed good calibration (Training: χ² = 3.77, P = 0.878; Validation: χ² = 6.20, P = 0.625). **(D)** Decision curve analysis in the validation cohort demonstrating clinical utility. The nomogram (purple line) provides superior net benefit compared to “treat all” (gray dashed) or “treat none” (black dotted) strategies across the clinical useful range (10%–75% threshold probability, shaded area). AUC, area under the curve; CI, confidence interval; χ², chi-square.

The performance of the final predictive nomogram was comprehensively evaluated in both the training and validation cohorts. The model demonstrated favorable discrimination in the training set, achieving an AUC of 0.865 (95% CI: 0.832-0.898). This high level of performance was successfully maintained in the independent validation set, which yielded an AUC of 0.842 (95% CI: 0.791-0.893), indicating the model’s good generalizability and minimal overfitting (**Figures 5A, B**). To explicitly demonstrate the added value of the core biomarker, an incremental value analysis was performed in the training cohort. Compared to a base clinical model that excluded homocysteine (AUC = 0.838, 95% CI: 0.802-0.874), the full model demonstrated a statistically significant improvement in discrimination (ΔAUC = 0.027, P = 0.015). Moreover, the addition of homocysteine yielded a continuous NRI of 0.354 (95% CI: 0.182-0.526, P < 0.001) and an IDI of 0.042 (95% CI: 0.018-0.066, P = 0.002), confirming a substantial reclassification benefit and an overall enhancement in predictive accuracy. Calibration plots for both cohorts showed reasonable agreement between the predicted probabilities and the actual observed frequencies of DR (**Figure 5C**). Beyond visual inspection and the non-significant Hosmer-Lemeshow tests (training P = 0.878, validation P = 0.625), modern quantitative metrics confirmed adequate calibration. In the training cohort, the optimism-corrected Brier score was 0.132, the CITL was 0.02, and the calibration slope was 0.96. In the independent external validation cohort, the model demonstrated a realistic level of transportability: the Brier score was 0.148, the CITL was 0.15 (indicating a slight systematic underestimation of risk), and the calibration slope was 0.89. The clinical utility of the nomogram was assessed using DCA. The DCA plot revealed that using the nomogram to inform clinical decisions provides a greater net benefit than either the “treat all patients” or “treat no patients” strategies across a wide and practical range of threshold probabilities, from approximately 10% to 75% (**Figure 5D**). This suggests that the nomogram is a valuable tool for improving clinical decision-making in identifying patients at high risk for diabetic retinopathy.

### 3.5 Subgroup analysis

**Figure 6:**
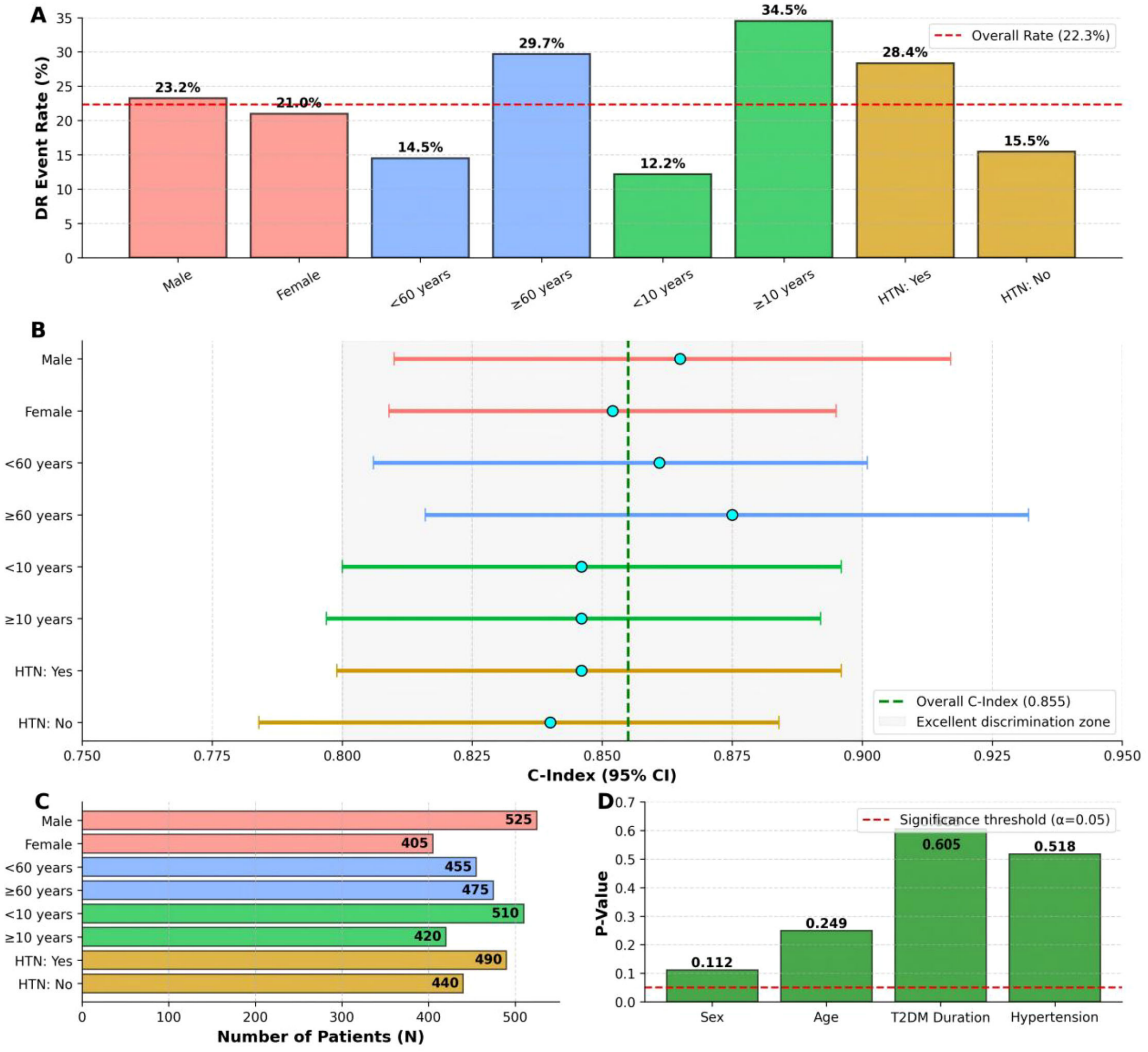
Comprehensive subgroup analysis of the nomogram’s predictive performance. The figure is composed of four panels providing a detailed evaluation across predefined subgroups. **(A)** Bar chart illustrating the event rate of diabetic retinopathy (DR) within each subgroup, with a dashed line indicating the overall event rate in the total cohort. **(B)** Forest plot displaying the C-index and its corresponding 95% confidence interval (CI) for the nomogram in each subgroup. A vertical dashed line represents the C-index for the entire cohort. **(C)** Horizontal bar chart showing the sample size distribution (number of patients, N) for each subgroup category. **(D)** Bar chart presenting the P-values for the interaction tests for each stratification variable, with a dashed line indicating the significance threshold (α = 0.05).

To further assess the stability and applicability of the nomogram, subgroup analyses were conducted based on key demographic and clinical characteristics across the entire cohort (N = 930). The performance of the nomogram was evaluated within subgroups defined by sex (male vs. female), age (<60 vs. ≥60 years), duration of T2DM (<10 vs. ≥10 years), and history of hypertension (yes vs. no). The nomogram maintained adequate and consistent discriminatory power across all analyzed subgroups, with C-indexes ranging from 0.840 to 0.875. Crucially, the tests for interaction yielded non-significant P-values for all subgroups (all P for interaction > 0.05). These results, visualized in **Figure 6**, indicate that the predictive performance of the model is stable and not significantly influenced by these baseline patient factors, thereby confirming its broad applicability.

## 4 Discussion

In this study, we successfully developed and validated a satisfactory nomogram for estimating the probability of prevalent diabetic retinopathy in patients with type 2 diabetes mellitus. The final model incorporated eight readily accessible variables: age, T2DM duration, systolic blood pressure, HbA1c, HDL-C, eGFR, UACR, and homocysteine. The nomogram demonstrated favorable discrimination in both the training (AUC = 0.865) and validation (AUC = 0.842) cohorts, along with adequate calibration and consistent performance across various patient subgroups. The findings suggest that this tool, which combines traditional risk factors with the metabolic marker homocysteine, may hold potential for clinical application in stratifying patient risk and guiding screening strategies.

The inclusion of established risk factors such as longer T2DM duration, elevated HbA1c, and higher systolic blood pressure aligns with a large body of existing evidence. These three factors represent the cornerstones of DR pathogenesis, reflecting cumulative glycemic burden and microvascular pressure, and their significance is consistently reported in landmark studies and meta-analyses (15, 16). Our model confirms their foundational role in assessing prevalent DR. Similarly, the inclusion of renal function markers, specifically a lower eGFR and a higher UACR, as independent indicators underscores the systemic nature of diabetic microvascular damage. Diabetic retinopathy and nephropathy are thought to share common pathophysiological pathways, including systemic inflammation, oxidative stress, and endothelial dysfunction, a concept often referred to as the “common soil” hypothesis (17). The strong association found in our model reinforces the clinical wisdom of concurrently screening for both complications, as the presence of one often signals an elevated risk for the other.

A key finding of this research is the identification of serum homocysteine as an informative and independent indicator of diabetic retinopathy. Homocysteine is a sulfur-containing amino acid, and elevated levels, or hyperhomocysteinemia, have been established as a risk factor for various vascular diseases. Its role in DR is increasingly recognized, with proposed mechanisms including the induction of profound oxidative stress on retinal endothelial cells, promotion of a pro-inflammatory state within the retinal microvasculature, and direct impairment of the blood-retinal barrier integrity (18, 19). By demonstrating its significance even after adjusting for traditional risk factors, our model elevates homocysteine from a mere association to a valuable addition to a clinical screening tool, suggesting that its measurement could enhance risk stratification protocols. Crucially, our incremental value analysis objectively quantified this contribution; the integration of homocysteine into the base clinical model yielded statistically significant improvements in discrimination (ΔAUC), reclassification (NRI), and overall predictive accuracy (IDI). However, while these statistical metrics demonstrate incremental value, the actual implementation benefit and broad transportability of including homocysteine in routine screening remain to be definitively established in diverse, real-world prospective cohorts. Furthermore, the model identified a lower HDL-C level as a significant risk factor, which is consistent with literature suggesting HDL-C has a protective role through its anti-inflammatory, antioxidant, and endothelial-protective properties (20).

When compared to other existing DR risk assessment models, our nomogram offers a distinct combination of clinical variables that is both comprehensive and clinically pragmatic. Many models rely primarily on glycemic and hypertensive markers (21), while ours integrates a key metabolic marker (homocysteine) and informative indicators of renal microvascular health (eGFR and UACR). The performance of our model, with an AUC of 0.842 in the validation set, appears numerically comparable or superior to several recently published nomograms whose AUCs typically range from 0.75 to 0.85 (22, 23). However, such comparisons should be interpreted cautiously. Direct comparisons of AUC values across different studies are inherently limited unless the study populations, specific outcome definitions (such as any-stage versus vision-threatening DR), candidate clinical variables, and validation strategies are highly similar. Therefore, while our model’s performance is favorable, its relative superiority must be strictly contextualized within these methodological constraints. The decision curve analysis provides a practical dimension to these performance metrics, demonstrating that the use of our nomogram yields a net benefit across a wide range of clinical risk thresholds (10% to 75%). This finding suggests the nomogram is not just statistically stable but also demonstrates potential clinical applicability, helping to identify higher-risk patients who would benefit most from intensified screening while avoiding unnecessary procedures for those at lower risk. Crucially, the intended clinical application of this model is specifically for screening prioritization in primary care or endocrinology settings. Rather than functioning as a prognostic model to predict future disease onset, the tool utilizes cross-sectional clinical data to estimate the current probability of prevalent DR, thereby guiding clinicians on which patients require an immediate, prioritized referral for a comprehensive ophthalmological examination.

This study has several strengths. The use of LASSO regression for variable selection is a sophisticated statistical method that minimizes overfitting and selects the most impactful variables from a large set of candidates. The comprehensive model evaluation, including discrimination, calibration, and the exploration of potential clinical utility via DCA, strictly adheres to contemporary best practices and reporting standards in clinical prediction model development and rigorous calibration assessment (24–26). Furthermore, the stability of the model’s performance confirmed through subgroup analysis suggests its potential applicability across diverse patient populations typically seen in a clinical setting. However, it is crucial to emphasize that while DCA suggests potential usefulness across certain threshold probabilities, these results are preliminary and hypothesis-generating. They do not, by themselves, establish that the model is definitively ready to guide routine screening protocols or immediately personalize clinical management without further prospective impact studies.

However, this study has several notable limitations that warrant a cautious interpretation of our findings. First, the retrospective, hospital-based design is inherently susceptible to unmeasured confounders and unresolved cohort design issues frequently encountered in real-world clinical databases. Second, the exclusion of 310 initially identified patients due to incomplete core laboratory records, such as missing plasma homocysteine or urinary albumin-to-creatinine ratio data, may have introduced a degree of selection bias. Although our formal comparative analysis demonstrated no significant demographic or metabolic differences between the included and excluded cohorts, this remains a potential constraint on the representativeness of the sample. Third, our sample size justification was based on the traditional events per variable rule of thumb rather than contemporary criteria considering expected shrinkage, model complexity, and target predictive performance, which might impact the precision of our model estimates. Fourth, the primary outcome was defined as a binary diagnosis of any-stage diabetic retinopathy rather than focusing on referable or vision-threatening stages. While this approach aligns with the screening objectives in primary care and endocrinology settings—where identifying any retinal microvascular complication is the priority—it inevitably merges clinically heterogeneous disease states and limits the model’s utility in predicting imminent vision loss. Fifth, our broad binary categorization of medication use fails to capture crucial pharmacological nuances such as specific drug classes, treatment intensity, and therapy duration, leaving room for residual confounding. Finally, the study was conducted within a narrow geographic setting in Shanghai. Given potential variations in ethnic backgrounds and clinical practice patterns, the transportability of our findings to broader populations remains to be confirmed through multi-regional prospective studies incorporating detailed severity grading and longitudinal data.

## 5 Conclusion

This study presents the development and validation of a nomogram integrating the metabolic biomarker homocysteine with established clinical and laboratory parameters for predicting diabetic retinopathy in patients with type 2 diabetes. Within our retrospective cohorts, the model demonstrated promising discriminatory ability and adequate calibration, suggesting its potential to assist in individualized risk assessment. However, given the retrospective nature of the data, the current findings should be interpreted cautiously. This model requires further rigorous validation in broader, prospective, and methodologically clearer settings to fully establish its definitive clinical utility before it can be routinely implemented to optimize screening protocols and personalize patient management.

## Data Availability

The raw data supporting the conclusions of this article will be made available by the authors, without undue reservation.

## Supplementary material

Supplementary Figure S1 Restricted cubic spline (RCS) curves evaluating the relationships between continuous predictors and the risk of diabetic retinopathy. The solid lines represent the estimated log-odds of diabetic retinopathy, and the shaded areas represent the corresponding 95% confidence intervals. The reference line (log-odds = 0, odds ratio = 1) is indicated by the horizontal dashed line. Knots were placed at the 10th, 50th, and 90th percentiles of each continuous variable. **(A)** Age, **(B)** T2DM Duration, **(E)** HDL-C, **(F)** eGFR, **(G)** UACR, and **(H)** Homocysteine demonstrated linear relationships (all P for non-linearity > 0.05). **(C)** Systolic Blood Pressure and **(D)** HbA1c showed mild non-linearity predominantly at the extreme tails (P for non-linearity < 0.05) but remained predominantly monotonic across their clinically relevant interquartile ranges. Likelihood ratio tests confirmed that incorporating non-linear terms did not significantly improve model fit, justifying their inclusion as continuous linear terms in the final parsimonious model.
