# Supplementary figures and images for: Development and validation of a multi-center nomogram for the presence of diabetic retinopathy in patients with type 2 diabetes: incorporating homocysteine, glycemic, lipid, and renal markers
Source: Front Endocrinol (Lausanne). 2026 Apr 22;17:1822839. doi: 10.3389/fendo.2026.1822839 (PMC13143537; doi:10.3389/fendo.2026.1822839)

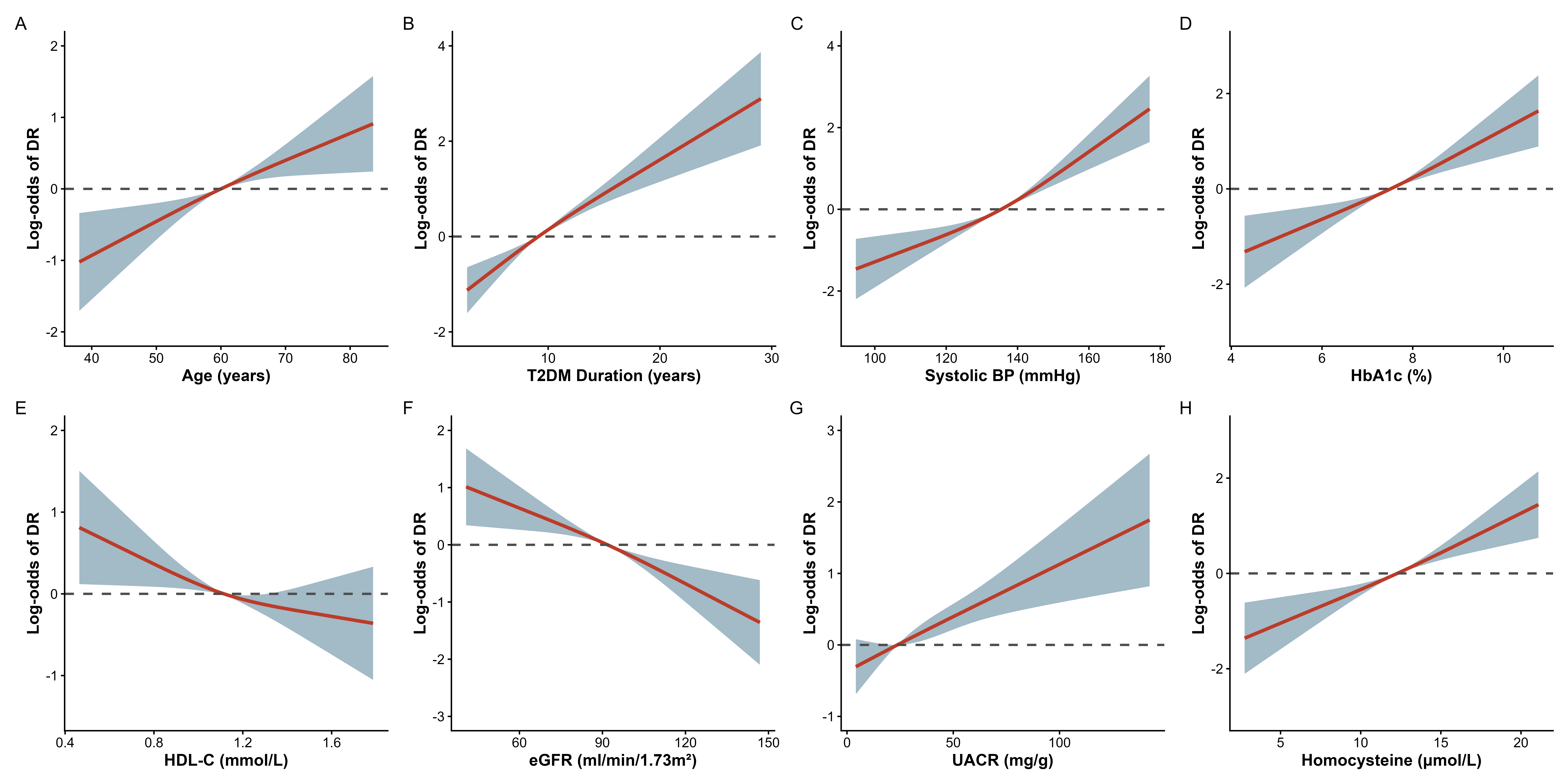

Supplement: Supplementary Figure S1 — Restricted cubic spline (RCS) curves evaluating the relationships between continuous predictors and the risk of diabetic retinopathy. The solid lines represent the estimated log-odds of diabetic retinopathy, and the shaded areas represent the corresponding 95% confidence intervals. The reference line (log-odds = 0, odds ratio = 1) is indicated by the horizontal dashed line. Knots were placed at the 10th, 50th, and 90th percentiles of each continuous variable. (A) Age, (B) T2DM Duration, (E) HDL-C, (F) eGFR, (G) UACR, and (H) Homocysteine demonstrated linear relationships (all P for non-linearity > 0.05). (C) Systolic Blood Pressure and (D) HbA1c showed mild non-linearity predominantly at the extreme tails (P for non-linearity < 0.05) but remained predominantly monotonic across their clinically relevant interquartile ranges. Likelihood ratio tests confirmed that incorporating non-linear terms did not significantly improve model fit, justifying their inclusion as continuous linear terms in the final parsimonious model. [file Image1.tiff]
