# Supplementary material for: Development and validation of a multi-center nomogram for the presence of diabetic retinopathy in patients with type 2 diabetes: incorporating homocysteine, glycemic, lipid, and renal markers
Source: Front Endocrinol (Lausanne). 2026 Apr 22;17:1822839. doi: 10.3389/fendo.2026.1822839 (PMC13143537; doi:10.3389/fendo.2026.1822839)
Supplement: Supplementary file 2 [file Table1.docx]

**Supplementary Table S1.** Comparison of baseline characteristics between the included study cohort and patients excluded due to incomplete core laboratory data

| **Characteristic** | **Included Cohort (n=930)** | **Excluded Cohort (n=310)** | **Statistic** | **P-value** |
| --- | --- | --- | --- | --- |
| Demographics |  |  |  |  |
| Age, years | 59.9 ± 10.0 | 60.6 ± 10.3 | t = -1.06 | 0.288 |
| Sex, Male, n (%) | 525 (56.5) | 168 (54.2) | χ² = 0.48 | 0.488 |
| T2DM Duration, years | 8.0 (5.0-12.0) | 7.5 (4.0-11.5) | U = 138120 | 0.185 |
| Anthropometric & Vitals |  |  |  |  |
| Body mass index, kg/m² | 26.2 ± 3.8 | 26.0 ± 4.0 | t = 0.78 | 0.435 |
| Systolic blood pressure, mmHg | 135.2 ± 18.1 | 134.2 ± 17.5 | t = 0.85 | 0.395 |
| Diastolic blood pressure, mmHg | 81.0 ± 10.4 | 80.5 ± 10.1 | t = 0.74 | 0.459 |
| Routine Laboratory Parameters |  |  |  |  |
| HbA1c, % | 7.5 ± 1.4 | 7.4 ± 1.3 | t = 1.15 | 0.250 |
| Fasting plasma glucose, mmol/L | 8.1 ± 2.5 | 8.0 ± 2.3 | t = 0.63 | 0.528 |
| Total cholesterol, mmol/L | 4.6 ± 1.1 | 4.5 ± 1.0 | t = 1.45 | 0.147 |
| Triglycerides, mmol/L | 1.8 (1.2-2.6) | 1.7 (1.2-2.5) | U = 139540 | 0.214 |
| LDL-C, mmol/L | 2.8 ± 0.9 | 2.7 ± 0.9 | t = 1.69 | 0.091 |
| HDL-C, mmol/L | 1.1 ± 0.3 | 1.1 ± 0.3 | t = 0.00 | 0.999 |
| eGFR, ml/min/1.73m² | 92.7 ± 23.0 | 93.5 ± 22.5 | t = -0.53 | 0.596 |

Notes: Data are presented as mean ± standard deviation for normally distributed continuous variables, median (interquartile range) for skewed continuous variables, and frequency counts with percentages (n, %) for categorical variables. The 'Included Cohort' consists of the final pooled sample utilized for model development and external validation. The 'Excluded Cohort' comprises patients who met all primary inclusion criteria but were excluded exclusively due to missing specialized core laboratory data (e.g., plasma homocysteine or urinary albumin-to-creatinine ratio). P-values were calculated using the independent samples Student's t-test for normally distributed continuous variables, the Mann-Whitney U test for non-normally distributed continuous variables, and the Pearson Chi-square test (χ²) for categorical variables. A P-value < 0.05 indicates statistical significance. The lack of significant differences across all comprehensive routine clinical and biochemical parameters demonstrates that the exclusion process did not introduce substantial systematic selection bias into the study population. Abbreviations: T2DM, type 2 diabetes mellitus; HbA1c, glycated hemoglobin; LDL-C, low-density lipoprotein cholesterol; HDL-C, high-density lipoprotein cholesterol; eGFR, estimated glomerular filtration rate.
